# Identification and Characterization of miRNAs in *Chondrus crispus* by High-Throughput Sequencing and Bioinformatics Analysis

**DOI:** 10.1038/srep26397

**Published:** 2016-05-19

**Authors:** Fan Gao, FangRu Nan, Wei Song, Jia Feng, JunPing Lv, ShuLian Xie

**Affiliations:** 1 School of Life Science, Shanxi University, Taiyuan 030006, China; 2 College of Shanxi Physical Technology, Taiyuan 030006, China

## Abstract

*Chondrus crispus*, an economically and medicinally important red alga, is a medicinally active substance and important for anti-tumor research. In this study, 117 *C. crispus* miRNAs (108 conserved and 9 novel) were identified from 2,416,181 small-RNA reads using high-throughput sequencing and bioinformatics methods. According to the BLAST search against the miRBase database, these miRNAs belonged to 110 miRNA families. Sequence alignment combined with homology searching revealed both the conservation and diversity of predicted potential miRNA families in different plant species. Four and 19 randomly selected miRNAs were validated by northern blotting and stem-loop quantitative real-time reverse transcription polymerase chain reaction detection, respectively. The validation rates (75% and 94.7%) demonstrated that most of the identified miRNAs could be credible. A total of 160 potential target genes were predicted and functionally annotated by Gene Ontology analysis and Kyoto Encyclopedia of Genes and Genomes analysis. We also analyzed the interrelationship of miRNAs, miRNA-target genes and target genes in *C. crispus* by constructing a Cytoscape network. The 117 miRNAs identified in our study should supply large quantities of information that will be important for red algae small RNA research.

The discovery of small RNAs that regulate diverse biological functions represents one of the most important findings in modern biology^1^. One special class of small RNAs are microRNAs (miRNAs), which are 21–25 nt long non-coding RNAs^2^ encoded by endogenous genes^3^. The primary miRNA transcript, pri-miRNA, has a typical stem-loop structure and is recognized and cleaved by a Dicer-containing protein complex to yield a special intermediate known as pre-miRNA^4^. In plant nuclei, pre-miRNA is cleaved by Dicer-like 1 protein to produce a duplex of two complementary intermediate RNAs^5^. One strand of the RNA, known as the mature miRNA, is incorporated into an RNA-induced silencing complex (RISC)^6^; the other strand is known as the star miRNA, or miRNA^*^, and is usually degraded^7^. After the formation of a complex via loading of miRNAs into the RISC, mRNA degradation or translational inhibition is controlled by binding between the miRNA-RISC complex and complementary target mRNA^8^.

Since their initial discovery as regulators in *Caenorhabditis elegans*, many miRNAs have been identified and demonstrated they play crucial roles in various plant and animal biological processes^9^. While miRNAs have been investigated and characterized in detail in many model organisms^10^, little is known in non-model plant organisms^11^. MiRNAs identification in non-model plants has recently been expedited by the rapid development of high-throughput sequencing (HiSeq) and bioinformatics techniques^12^,^13^. A number of new or conserved miRNAs have been found in plants such as maize, potato, wheat, tobacco, rice and sorghum^11^,^14^,^15^,^16^,^17^,^18^. In addition, the large amount of genomic and other sequence data available in biological databases provides important, comprehensive support for research on miRNAs in non-model plants^19^.

*Chondrus crispus* is an economically and medicinally important marine red alga that grows mainly on rocks along the North Atlantic coast^20^. At present, it has been planted and processed in some coastal countries, such as United States, Philippines, Indonesia, China and Ireland^21^,^22^. Most biological studies of *C. crispus* have focused on photosynthesis, genetic variation, bioremediation, bioactive components and mRNA expression mechanisms^23^,^24^,^25^,^26^,^27^. Carrageenan, a polysaccharide mixture mainly composed of sulfate, galactose and its derivatives^28^,^29^, was initially extracted from *C. crispus* growing along the southern coast of Ireland in 18^th^ century. This substance has been recently used as a food additive as well as the main source of algal polysaccharide, which has valuable medicinal and health-related properties, such as tumor and virus inhibition, hypertension and hyperlipemia prevention, control of hyperglycemia, immunity enhancement, and antioxidant, antibacterial and anti-inflammatory activities^30^,^31^,^32^,^33^,^34^,^35^,^36^,^37^,^38^,^39^,^40^,^41^. Notably, this unique anti-tumor effect may be the main reason for interest in this red alga. Some research on the basis of the anti-tumor activity of this algal polysaccharide—of interest for use in tumor treatment—has been reported. For instance, Zhou found that seaweed polysaccharide extracted from λ-carrageenan had a significant inhibitory effect on both tumor cell lines (YAC-1, HeLa and H-7402) cultured in *vitro* as well as tumor cells (S180 and H-22) cultured in *vivo* in ascites cells^36^. To date, however, the detailed inhibitory process and gene regulatory mechanism related to the effect of algal polysaccharide is little known. Even so, two different hypotheses regarding a possible mechanism have been proposed. One is that the antitumor activity occurs via direct inhibition on the molecular level—that is, only the polysaccharide with its unique molecular weight and structure can combine with the target cancer cell to inhibit its growth after a series of signal transfer processes^40^,^41^,^42^,^43^,^44^. The other view is that of indirect inhibition on the immune level: the polysaccharide inhibits tumors by enhancing the body’s immunity, mainly by improving the body’s antioxidant activity^40^,^45^,^46^,^47^,^48^. Whether there is a new anti-tumor way at the cellular level based on deep sequencing in *C. crispus*, what miRNAs and how they play regulation roles in the process, which still needs to explore. Identification and characterization of miRNAs in *C. crispus* will be the essential first step in these tasks.

Compared with the rapidly increasing amount of information available for miRNAs in model and advanced plants^49^,^50^,^51^,^52^, little is known about miRNAs in the non-model lower seaweed *C. crispus*. Although over 35,000 miRNAs from 223 species have been submitted to the miRNA database (miRBase 21.0, June 2014; http://www.mirbase.org/)^53^, no miRNAs from *C. crispus* have been deposited. Fortunately, the establishment of next-generation high-throughput sequencing (HiSeq) technology^54^ combined with the recent development of many bioinformatics tools^55^,^56^,^57^ and the availability of *C. crispus* genome sequences^58^ have facilitated the identification of miRNAs in *C. crispus*. Such identification will be extremely important to comprehensively understand the diverse biological regulatory processes mediated by miRNAs in *C. crispus*. Investigation of miRNAs in *C. crispus*, such as examination of whether and how they contributes to tumor and cancer cell growth inhibition, may be illuminating to explore an effectively approach to treatment of cancer in future.

## Results

### HiSeq of small RNAs in *C. crispus*

**Figure 1:**
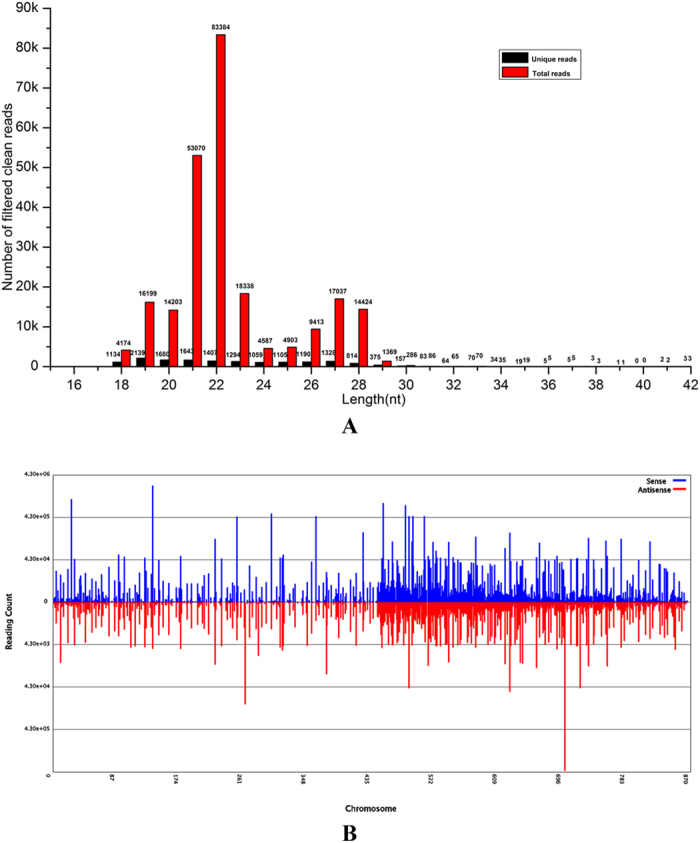
Size distribution and positional distribution of small RNAs in *Chondrus crispus.* (**A**) Sequencing clean reads distribution with removed t/r/sn/snoRNAs in the small-RNA library. As shown by their unique and total numbers, 22- and 21-nt length reads were the most abundant. (**B**) Distribution of sequencing reads on the *C. crispus* chromosomes. Abundant of reads were distributed between contig435–contig870, with the highest count of small RNAs on the antisense strand around contig705.

A small-RNA population isolated from total RNA of *C. crispus* was subjected to HiSeq. As shown in Table 1, a total of 11,243,850 clean reads were obtained. The numbers and proportions of small RNAs mapped to the *C. crispus* genome are given in Table 2. As shown in Fig. 1A, most small RNAs with removed t/r/sn/snoRNAs were 22 nt (9.01% unique reads and 34.51% total reads) in length followed by 21 nt (10.52% unique reads and 21.96% total reads). According to the simulated chromosomes distribution of small RNAs illustrated in Fig. 1B, most small RNAs were distributed on either strand at contig435–contig870, with the highest number of small RNAs distributed on the antisense strand around contig705. After searching different nucleic acid databases, we were only able to annotate less than half of small RNAs (Fig. S1A,B), with 6,003,013 unannotated reads (266,364 unique reads) still requiring further analysis. In spite of this, 241,681 of the small-RNA reads (15,614 unique reads) could still be annotated as known miRNAs. After removing repeat sequences, 106 annotated known miRNAs were identified in *C. crispus* (Table S1).

### Identification of novel and additional conserved miRNAs in *C. crispus*

In addition to mapping unannotated reads to *C. crispus* genomic exon antisense strand, intron and intergenic regions, we subjected the candidate unannotated reads to a series of screening assays using stringent identification criteria (see Materials and Methods). As a result, nine specific or novel miRNAs were identified (Table S1). Partial secondary structures of the precursors (i.e., canonical hairpin structures) are shown in Fig. S2A–F. Besides the sequencing data, we were able to use rich (4,120) EST assemblies of *C. crispus* to predict additional potential conserved miRNAs. For this purpose, we attempted to align these ESTs to all plant miRNAs deposited in miRBase. Using the plant miRNA criteria described in Materials and Methods, only two potential miRNAs were identified as conserved miRNAs with high confidence (Table S1). Consequently, a total of 117 miRNAs—including 108 conserved (106 from HiSeq and 2 from ESTs) and 9 novel ones—were identified in *C. crispus*. The length distribution of identified miRNAs ranged from 18 to 23 nt, with a highest-frequency length category being 19 nt (Fig. S3A). Nucleotide frequencies and first-nucleotide biases of novel miRNAs are shown in Fig. S3B,C, respectively. As revealed in Fig. S3B, the novel miRNAs were biased toward C, U and G in *C. crispus*. The first 5′-end nucleotide of novel miRNAs were biased toward G and U (Fig. S3C).

### Conservation and diversity of *C. crispus* miRNA families

**Figure 2:**
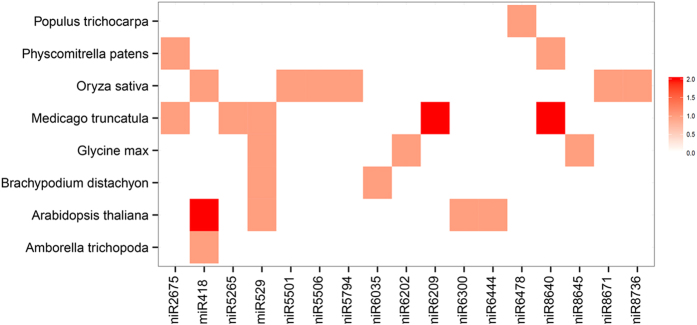
Distribution of conserved miRNA families in different plant species. Data for mature miRNAs in other plant species are from miRBase21.0. Color coding is used to indicate the number of miRNA members, with dark red corresponding to the highest number and white the lowest.

To investigate potential conserved miRNA families, we performed a nucleotide alignment and cluster analysis. Altogether, 110 miRNA families comprising 117 members were predicted in *C. crispus* (Table S2). As shown in Table 3, most predicted families had only one member, with the largest family containing four. To research the conservation and diversity of *C. crispus* miRNA families in plants, we aligned 17 randomly selected conserved miRNA families against plant miRNA families in miRBase (Table S3). As shown in Fig. 2, these 17 families were close homologs to those in eight plant species, indicating that their potential biological functions may be relatively conserved. Among the 17 miRNA families in *C. crispus*, 7 and 6 families had homologs in *Medicago truncatula* and *Oryza sativa*, respectively. The miR529 family predicted in *C. crispus* had homologs in *Arabidopsis thaliana*, *Brachypodium distachyon*, *Glycine max* and *Medicago truncatula*, indicating that this miRNA family may have descended from the same common sequence in an ancestral species. On the basis of the known annotations of these plant species, we may be able to predict the functions of *C. crispus* miRNAs. Moreover, the distribution of these miRNA families in plants, including bryophytes, gymnosperms and angiosperms, demonstrates their diversity.

### Validation of miRNAs of *C. crispus*

**Figure 3:**
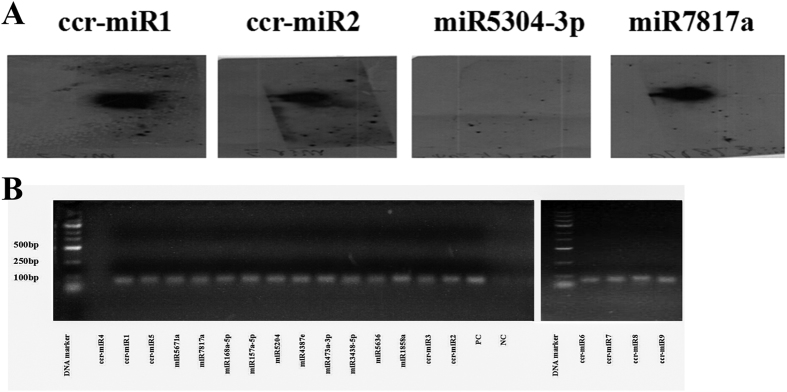
Gel-based detection of *Chondrus crispus* miRNAs. (**A**) RNA gel blot hybridization of digoxigenin-labeled probes for four *C. crispus* miRNAs. (**B**) Agarose gel of stem-loop qRT-PCR products based on 19 *C. crispus* miRNAs. PC stands for the positive controls, and NC stands for the negative controls. U6 was used as PC, and double distilled water was used as NC.

To validate the credibility of HiSeq and bioinformatics analysis results, we randomly selected four miRNAs and subjected them to northern blot detection. The oligonucleotides used are listed in Table S4. Three of the four miRNAs were detected by northern blotting (Fig. 3A). In addition, 19 miRNAs including all 9 novel miRNAs and 10 randomly selected conserved miRNAs were subjected to stem-loop quantitative real-time reverse transcription (qRT) PCR validation. The qRT-PCR primers used are listed in Table S5. Eighteen of the 19 miRNAs were detectable by qRT-PCR (Fig. 3B). The results produced by the two validation methods confirm that most miRNAs identified in this study are credible.

### Target gene prediction and functional analysis of *C. crispus* miRNAs

Plant miRNAs can bind almost perfectly to their target genes via typical complementarity matching and regulate the mRNA post-transcriptional process by transcript degradation or translational inhibition. To better understand the biological role of miRNAs in *C. crispus*, we used TargetFinder to predict putative miRNA targets, with *Chondrus* ESTs and transcripts as a reference. As shown in Table 4, 160 target genes were predicted. Among them, 156 target genes were predicted according to mRNA-conserved miRNA sequence complementarity and four were identified on the basis of mRNA-novel miRNA sequence complementarity. As shown in Table S6, 32 of 160 targets were predicted to be potential translational inhibition regions, which indicate that miRNA translational inhibition may not be a typical inhibition mode in *C. crispus*. Certainly, we may underestimate the level of miRNA-mediated translational inhibition as a result of limitation of spatial and temporal analysis on target genes expression.

**Figure 4:**
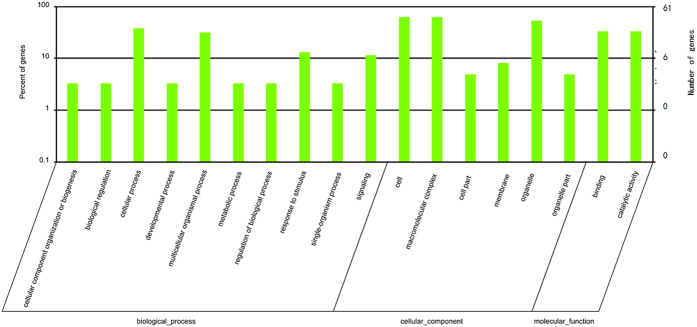
Gene Ontology (GO) classification of target genes. The x-axis shows the diverse biological functions of target genes according to three GO categories (biological process, cellular component and molecular function). The y-axis shows the percentage and number of these target genes.

According to GO classifications, the BP category contained the largest number of target genes (Fig. 4). The topological relationships of the 10 most enriched GO terms according to BP, CC and MF classifications are illustrated in the DAG in Fig. S4A–C. As indicated by Table S7, the three most highly enriched GO terms in biological process (BP), cellular component (CC) and molecular function (MF) were related to protein folding, intracellular membrane-bounded organelles and nucleotide binding, respectively. The observed enrichment of GO terms may provide some insights into important biological processes in *C. crispus*, such as metabolism, development and stress response.

**Figure 5:**
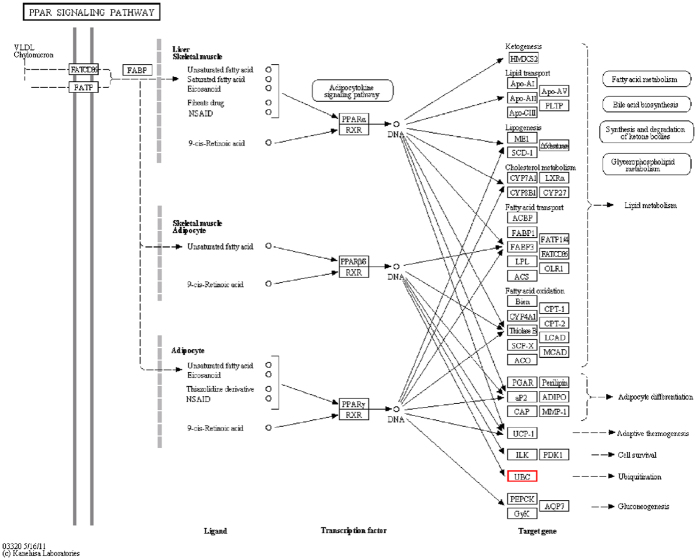
Peroxisome proliferator-activated receptor (PPAR) signaling pathway. The PPAR signaling pathway enriched in miRNA target genes according to the KEGG analysis. Small boxes represent proteins or enzymes and red ones indicate the candidate target genes encoding them. The small circles represent metabolites. The arrows represent different metabolic pathways. The detailed introduction about this pathway can be seen online. The website of it is: http://www.kegg.jp/entry/rno03320.

KEGG mapping can be used to analyze target gene products and functions during metabolic processes. As shown in Table S8, Fig. 5 and Fig. S5, the peroxisome proliferator-activated receptor (PPAR) signaling pathway was the most highly enriched pathway in *C. crispus*, with 22 relevant target genes. As the most enriched target gene, the ubiquitin gene *ubiquitin C* may play an essential role in this signaling pathway in *C. crispus*.

Finally, we generated a Cytoscape network among miRNAs, miRNA-target gene and target genes. Major enriched and cross-linked networks are shown in Fig. S6. The most highly enriched network was miR6145 with 26 relevant target genes. MicroRNAs miR5769, miR6485, miR9484 and miR5304 and 11 relevant target genes formed the most extensive cross-linked network.

## Discussion

*Chondrus crispus* is one of the world’s most economically and medically important red alga. Prior to the present study, however, fundamental knowledge of miRNA sequences and functions has been lacking for *C. crispus*. In this investigation, we applied HiSeq and bioinformatics analyses to identify and characterize miRNAs in *C. crispus*. The 117 miRNAs identified in our study should supply large quantities of information that will be important for red algae small RNA research. The identified miRNAs should also help fill the miRNA knowledge gap in this exotic red alga and serve as the foundation for further research on its medicinal mechanisms.

Some miRNAs are expressed in different tissues or at different times^59^. This spatial and temporal variation in gene expression may be the major limiting factor in validation of miRNAs. However, we can not ignore the effects of other validation methods on genes expression such as situ hybridization except northern blot and stem-loop qRT-PCR. Using northern blot and stem-loop qRT-PCR methods, we were able to validate 3 out of 4 and 18 out of 19 miRNAs, respectively. As suggested by the mature miRNA sequence on the 3′-end of its precursor, randomly selected miR5304-3p may be a star sequence, with consequent low expression that may be difficult to detect (Fig. 3A). Except for ccr-miR4, however, all novel miRNAs and 10 conserved miRNAs identified in our study were detected by stem-loop qRT-PCR (Fig. 3B), demonstrating that these miRNAs identified by HiSeq and bioinformatics should be credible. Next, the spatial and temporal analysis on target genes will be necessary.

Conservation and diversity analysis of predicted miRNA families in various plant species demonstrates that a potential species-species miRNA link may exist^49^. Further study is needed to determine whether this link is related to biological evolution.

The predicted target genes and the functions of their products provide useful clues for research on some essential biological processes in *C. crispus*. According to GO analysis^60^, for example, the enriched target genes (GO: 0016070) regulated by miR856-5p in BP (Tables S6 and S7) are associated with an essential RNA metabolic process. Several questions remain to be resolved, namely, the identity of the RNA metabolic process, the metabolic regulatory mechanism, and the nature of miRNA participation in this process. One interesting finding is that the PPAR signaling pathway (KO ID: K08770) was the most enriched pathway according to the KEGG analysis (Table S8 and Fig. S5)^61^, with the most enriched target gene being the essential gene *ubiquitin C* that has been cloned in many species^62^,^63^,^64^. Research has demonstrated that this gene encodes a polyubiquitin precursor, which would lead to increased ubiquitin-based effects within a human cell, such as cell cycle regulation, kinase modification, endocytosis and regulation of other cell signaling pathways^65^. Increased ubiquitin is a potential cause of tumor induction. Consequently, enhancement of deubiquitination must be very important for tumor inhibition and may be yet another potential anti-tumor pathway operating at the cellular level in *vivo*. In addition to the unique algal polysaccharide in *C. crispus*, we speculate that inhibiting or blocking the post-transcription process of *ubiquitin C* may be another effective supplement for anti-tumor therapy in future. Moreover, the Cytoscape network will point out the direction for further study on this gene expression process.

## Methods

### Plant material

Material was obtained from eight tissues collected from four different *C. crispus* samples at the asexual diploid sporophyte stage. All tissues were sheared into 2-cm cubes taken from two different locations on each sample. The four samples were collected on 1 May, 2014 on the southeastern coast of Qingdao near the Yellow Sea (about 36° N latitude and 120° E longitude) by researchers from the Institute of Oceanology of the Chinese Academy of Sciences (Qingdao, China). To discover as many miRNAs as possible, approximately 80-mg tissues were mixed together quickly as one sequencing sample pool for RNA extraction, immediately frozen in liquid nitrogen and stored at −80 °C.

### Total RNA isolation, small-RNA library construction and sequencing

After the high-quality extraction of total RNA from collected tissues using Trizol reagent (Invitrogen, Carlsbad, CA, USA), a small-RNA library was constructed from 10 μg of small RNA that had been isolated with a TruSeq Small RNA Sample Prep kit (Illumina, San Diego, CA, USA). Library quality assessment was performed on an Agilent 2100 Bioanalyzer (Agilent, Palo Alto, CA, USA) and a StepOnePlus Real-Time PCR system (ABI, Carlsbad, CA, USA). Sequencing was performed on an Illumina HiSeq 2000 instrument. The raw sRNA sequencing data has been uploaded to the SRA database in NCBI with the accession numbers: SRP066538/SRS1172958/SRX1445023/SRR3228749.

### Bioinformatics analysis of detected miRNAs

#### Standard bioinformatics analysis of small RNAs

**Figure 6:**
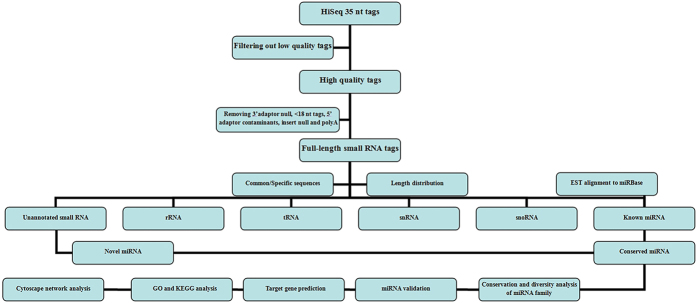
Flowchart of experiment and information analysis in *Chondrus crispus.*

After sequencing, we identified the clean reads by filtering out low-quality tags, poly (A) tags, and tags with 3′ adaptor nulls, insert nulls, 5′ adaptor contaminants, or fewer than 18 nt (Fig. 6). The resulting clean reads were annotated by alignment against *C. crispus* genome sequences in GenBank (as of June 2014) using the SOAP program (http://soap.genomics.org,cn/)^66^ and against all plant miRNAs in miRBase 21.0 (as of June 2014) using the Basic Local Alignment Search Tool (BLAST)^67^. After removal of known miRNAs, snRNAs, snoRNAs, rRNAs and tRNAs identified in miRBase 21.0, GenBank and Rfam 11.0 (http://rfam.sanger.ac.uk) databases^68^, the only remaining reads may be unannotated small RNAs. The yes/no decision tree of reads screening can be seen in Fig. S7.

#### Identification of novel miRNAs

To retrieve novel miRNAs, the unannotated small-RNA tags were first mapped to red alga genomic exon antisense strand, intron and intergenic regions using Mireap software (http://sourceforge.net/projects/mireap/)^69^. The small RNA tags mapped to these regions were then screened according to the following criteria^59^,^70^: (i) the precursor could form a perfect stem-loop structure; (ii) the miRNA and miRNA* formed a duplex with no more than two nucleotides on 5′- and 3′-end overhangs; (iii) no loops and bulges larger than 4 nt were located within the miRNA-miRNA* duplex and (iv) the value of the precursor’s minimum free energy of folding (MFE) was no more than −18 kcal/mol. Finally, the remaining highly credible sequences were retained as potential novel miRNAs (Fig. S1). Secondary structures of qualified pre-miRNAs (precursors) were then generated using Mireap.

#### Identification of additional conserved miRNAs

To uncover as many miRNAs as possible in *C. crispus*, we identified additional potential conserved miRNAs by BLASTn alignment of the 4,120 *C. crispus* expressed sequence tags (ESTs) in GenBank (as of June 2014) against 6,992 plant miRNAs and 8,450 precursors in miRBase 21.0. To be considered as a potential conserved miRNA, we used the following matching criteria^71^: (i) base-pairing between miRNA and miRNA* had no more than three mismatches; (ii) the length of the mature miRNA was 18–25 nt; (iii) candidates could not be identified as a rRNA, tRNA or other non-miRNA and (iv) the precursor of the miRNA had a perfect stem-loop secondary structure. In this study, additional conserved miRNAs and known miRNAs were both designated as conserved miRNAs.

#### Prediction of miRNA families

To predict potential miRNA families in *C. crispus*, conserved and novel miRNA sequences were aligned with Clustal X 1.83^72^ and used to construct a neighbor-joining phylogenetic tree with 1,000 replicates in MEGA 5.0 (http://www.megasoftware.net)^73^. Only miRNA sequences with homology percentages no less than 98% and with no more than two mismatches between them were considered to belong to the same miRNA family. In addition, sequences of 17 predicted conserved miRNA families were randomly selected and aligned to sequences from miRNA families of plant species deposited in miRBase. This alignment revealed the conserved nature and diversity of *C. crispus* miRNA families in different plant species.

### Validation of identified miRNAs

#### Northern blot validation

Four identified miRNAs, two conserved and two novel, were randomly selected and subjected to northern blot validation^74^. Enriched small-RNA samples (10 μg) were from our samples pool used to the deep-sequening. They were resolved on a 15% denaturing polyacrylamide gel and electrostatically transferred to Hybond-N+ nylon membrane (Amersham Biosciences, London, UK). Blot hybridization was carried out with miRNA-complementary DNA oligonucleotides labeled with digoxigenin (Roche, Basel, Switzerland). After a pre-hybridization incubation at 62 °C for 2 h, hybridization was performed with incubation at 42 °C for 18 h. Membranes were washed twice, first with 2× saline sodium citrate (SSC)/0.1% sodium dodecyl sulfate (SDS) for 5 min at room temperature and then with 0.5× SSC/0.1% SDS for 15 min at 65 °C. After incubation with blocking and antibody buffers for 30 min each at room temperature, membranes were immersed in detection buffer for 5 min. Following addition of 1 mL CDP-Star (a chemical luminescence material with the molecular formula C_18_H_19_Cl_2_Na_2_O_7_P), membranes were incubated at 25 °C for 5 min and 37 °C for 10 min, and then exposed to X-ray film for 20 min at 25 °C.

#### QRT-PCR validation

To further verify our identification results, 19 randomly chosen sequences, including all 9 novel miRNAs as well as 10 conserved ones, were subjected to stem-loop qRT PCR^75^. Stem-loop qRT-PCR amplifications were carried out using universal primer, RT primer and forward primer. In the first step, reverse transcription was performed using a HiScript 1st Strand cDNA Synthesis kit (Vazyme Biotech, NY, USA) in 20-μL reaction volumes consisting of 1 μg RNA, 0.5 μL RT primer, 5 μL of 2× RT Mix, 1 μL RT Enzyme Mix and RNase-free double-distilled water. Reaction conditions were 25 °C for 5 min, 42 °C for 20 min, 85 °C for 10 min and 4 °C for 5 min. In the second step, QRT-PCR was carried out in reaction mixtures comprising 1 μL cDNA, 0.5 μL universal primer, 0.5 μL forward primer, 5 μL of 2× Taq PCR Master Mix (Vazyme Biotech) and 3 μL distilled water. The qRT-PCR protocol was as follows: 95 °C for 5 min, followed by 35 cycles of 95 °C for 15 s, 60 °C for 30 s and 72 °C for 15 s, with a final step of 72 °C for 5 min and then 4 °C for 2 min. The resulting PCR products were detected by 1.5% agarose gel electrophoresis.

### Target gene prediction and functional analysis

#### Target gene prediction

Prediction of miRNA target genes was performed using TargetFinder 1.6 software (August 2010; http://carringtonlab.org/)^76^. All identified miRNAs were queried against the protein-coding gene database. We used transcript and EST sequences of *Chondrus* deposited in GenBank for target gene prediction. Based on homology between miRNAs and target transcripts, target sequences with the following characteristics were retained^49^: (i) no more than four mismatches, with no more than one mismatch in positions 1–9 and no mismatches at positions 10 and 11; (ii) no deletions or insertions; (iii) a perfect duplex at positions 8–12; (iv) no loops or bulges in either strand; (v) overhangs on 5′ and 3′ ends of no more than one nucleotide and (vi) a MFE value less than −18 kcal/mol between the miRNA and its complementary sequence. In addition, potential translational inhibition was predicted on the basis of whether a mismatch could be detected in the central complementary region of the miRNA sequence (9–11 nt) using psRNATarget (http://plantgrn.noble.org/psRNATarget/)^77^.

#### Gene Ontology (GO) functional classification and enrichment analysis

Before target gene functional classification, we analyzed GO functions using the following equation^78^:


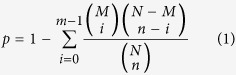


In this equation, *N* is the number of GO-annotated genes, *n* is the number of target gene candidates in *N*, *M* is the number of genes assigned to a particular GO term and *m* is the number of target gene candidates in *M*. GO terms with Bonferroni-corrected *p*-values ≤ 0.05 were defined as significantly enriched in target gene candidates. Functional classification of miRNA-regulated target genes was performed using the AmiGO tool on the Gene Ontology Consortium website (http://geneotology.org/) according to GO-controlled vocabularies that describe gene products in terms of BP, CC and MF^79^. Enriched GO terms and their topological structures were obtained using the Goseq package^80^. A directed acyclic graph (DAG) of the top 10 enriched terms based on the three above-mentioned ontologies was generated. Because of their scattered functional distribution, GO functional classification and enrichment analysis of target genes regulated by novel miRNAs could not be performed effectively.

### Kyoto Encyclopedia of Genes and Genomes (KEGG) metabolic pathway analysis

Various biological functions are cooperatively regulated by multiple genes. The main metabolic pathway associated with related target genes can be predicted by KEGG enrichment analysis. In this study, predicted target genes were assigned KEGG Orthology (KO) IDs based on homology and similarity of functional products using KOBAS 2.0 (http://kobas.cbi.pku.edu.cn/home.do)^81^. The KEGG pathway enrichment analysis used the same equation described above for the GO analysis, except that *N* was defined as the number of KEGG-annotated genes and *M* was the number of genes annotated to a certain pathway. Genes with a false discovery rate ≤ 0.05 were considered to be significantly enriched in target gene candidates. Using the results of the KEGG enrichment analysis, a reference KEGG metabolic pathway map was constructed with KegSketch software (http://genome.jp/kegg/)^82^ and significantly enriched pathway maps were generated. As in the GO analysis, KEGG pathway analysis could not be effectively performed on target genes regulated by novel miRNAs.

### Cytoscape network analysis

To investigate interrelationships among miRNAs, miRNA-target gene and target genes in *C. crispus*, a Cytoscape network was constructed with Cytoscape software (http://www.cytoscape.org)^83^. The network was analyzed to identify interactions of miRNAs, miRNA-target gene and target genes in *C. crispus*.

## Additional Information

**How to cite this article**: Gao, F. *et al.* Identification and Characterization of miRNAs in *Chondrus crispus* by High-Throughput Sequencing and Bioinformatics Analysis. *Sci. Rep.* **6**, 26397; doi: 10.1038/srep26397 (2016).

## Supplementary Material

Supplementary Figure

Supplementary Table S1

Supplementary Table S2

Supplementary Table S3

Supplementary Table S4

Supplementary Table S5

Supplementary Table S6

Supplementary Table S7

Supplementary Table S8

## Figures and Tables

**Table 1:** Summary of sequencing reads from a *Chondrus crispus* small-RNA library.

Type	Reads	Percent (%)
Total reads	11643105	
High quality	11601770	100%
3′adapter null	751	0.01%
Insert null	7634	0.07%
5′adapter contaminants	11613	0.10%
Smaller than 18nt	337810	2.91%
PolyA	112	0.00%
Clean reads	11243850	96.91%

**Table 2:** Summary of small RNAs mapped to the *Chondrus crispus* genome.

	Unique reads	Percent (%)	Clean reads	Percent (%)
Total small RNAs	3017665	100%	11243850	100%
Mapping to genome	153264	5.08%	4925432	43.81%

**Table 3:** Number of members of each miRNA family identified in *Chondrus crispus.*

Size of miRNA families	Number of miRNA families	Percent of conserved miRNA families (%)	Percent of novel miRNA families (%)
1	103	87.27%	6.36%
2	5	3.63%	0.00%
3	1	0.00%	1.82%
4	1	0.91%	0.00%

**Table 4:** Summary of predicted miRNA targets in *Chondrus crispus.*

miRNA type	miRNA number	Target gene number	Count of (miRNA::corresponding target gene)	Target location number
Conserved miRNA	45	156	160	160
Novel miRNA	2	4	4	4
Total	47	160	164	164
