# Supplementary material for: Identification and Characterization of miRNAs in Chondrus crispus by High-Throughput Sequencing and Bioinformatics Analysis
Source: Sci Rep. 2016 May 19;6:26397. doi: 10.1038/srep26397 (PMC4872230; doi:10.1038/srep26397)
Supplement: Supplementary Figure [file srep26397-s1.pdf]

Identification and Characterization of miRNAs in *Chondrus crispus* by High-Throughput Sequencing and Bioinformatics Analysis

Fan Gao[1], FangRu Nan[1], Wei Song[2], Jia Feng[1], JunPing Lv[1], ShuLian Xie[1,*]

[1]School of Life Science, Shanxi University, Taiyuan 030006, China

[2]College of Shanxi Physical Technology, Taiyuan 030006, China

Supplementary Figure Legends

Figure S1. Pie chart of small RNA distribution in *Chondrus crispus*

(A) Frequency distribution of unique small RNAs in *C. crispus.* (B) Frequency distribution of total small RNAs in *C. crispus.*

Figure S2. Secondary structure of predicted pre-miRNAs in *Chondrus crispus*

(A) Stem-loop structure of the conserved miRNA precursor miR169b in *C. crispus.* (B) Stem-loop structure of the novel miRNA precursor ccr-miR8 in *C. crispus.* (C) Stem-loop structure of the novel miRNA precursor ccr-miR1 in *C. crispus.* (D) Stem-loop structure of the novel miRNA precursor ccr-miR2 in *C. crispus.* (E) Stem-loop structure of the conserved miRNA precursor miR7817a in *C. crispus.* (F) Stem-loop structure of the conserved miRNA precursor miR5304-3p in *C. crispus.*

Figure S3. Size distribution of miRNAs in *Chondrus crispus*

(A) Percentage of predicted miRNAs according to length in *C. crispus*. The most abundant miRNAs were 19 nt long. (B) Percentage of nucleotide bias in novel miRNAs in *C. crispus.* Novel

miRNAs were biased toward C, U and G. (C) Percentage of first-nucleotide bias in novel miRNAs in *C. crispus*. The novel miRNAs were biased toward G and U.

Figure S4. Directed acyclic graph (DAG) of the 10 most enriched Gene Ontology (GO) terms

The DAG shows the topological relationships of the 10 most enriched GO terms. Circles and boxes with different colors represent enriched GO terms, in biological process, cellular component and molecular function categories. (A) DAG of enriched terms in the biological process category. (B) DAG of terms enriched for cellular components. (C) DAG of terms enriched for molecular function.

Figure S5. Chart of KEGG pathways enrichment analysis

Top 20 statistics of KEGG pathways enrichment were shown based on two rich factors: target gene number and Q-value. Circular mark is used to indicate the number of genes enriched in the pathway, with the biggest one corresponding to the most genes and smallest one the fewest. Color coding is used to indicate the Q-value of pathway, with dark blue corresponding to the minimum value and light blue the maximum. Based on the two factors, PPAR signaling pathway is the most significant pathway in *C. crispus*.

Figure S6. Subset of the Cytoscape network constructed for *Chondrus crispus*

The Cytoscape network reflects the relationships among predicted miRNAs, miRNA-target genes and target genes in *C. crispus.*

Figure S7. Decision tree of small RNAs in *Chondrus crispus*

The yes/no decision tree clearly shows the conditions used to process and characterize the reads including how these reads selected as novel microRNAs or conserved ones.

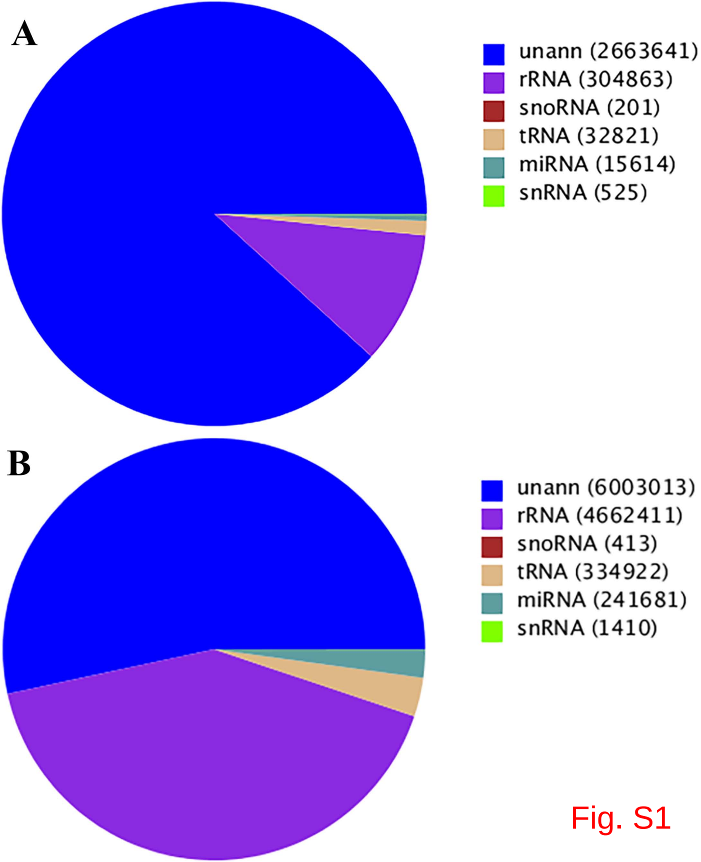
A
unann (2663641)
rRNA (304863)
snoRNA (201)
tRNA (32821)
miRNA (15614)
snRNA (525)
B
unann (6003013)
rRNA (4662411)
snoRNA (413)
tRNA (334922)
miRNA (241681)
snRNA (1410)

Fig. S1

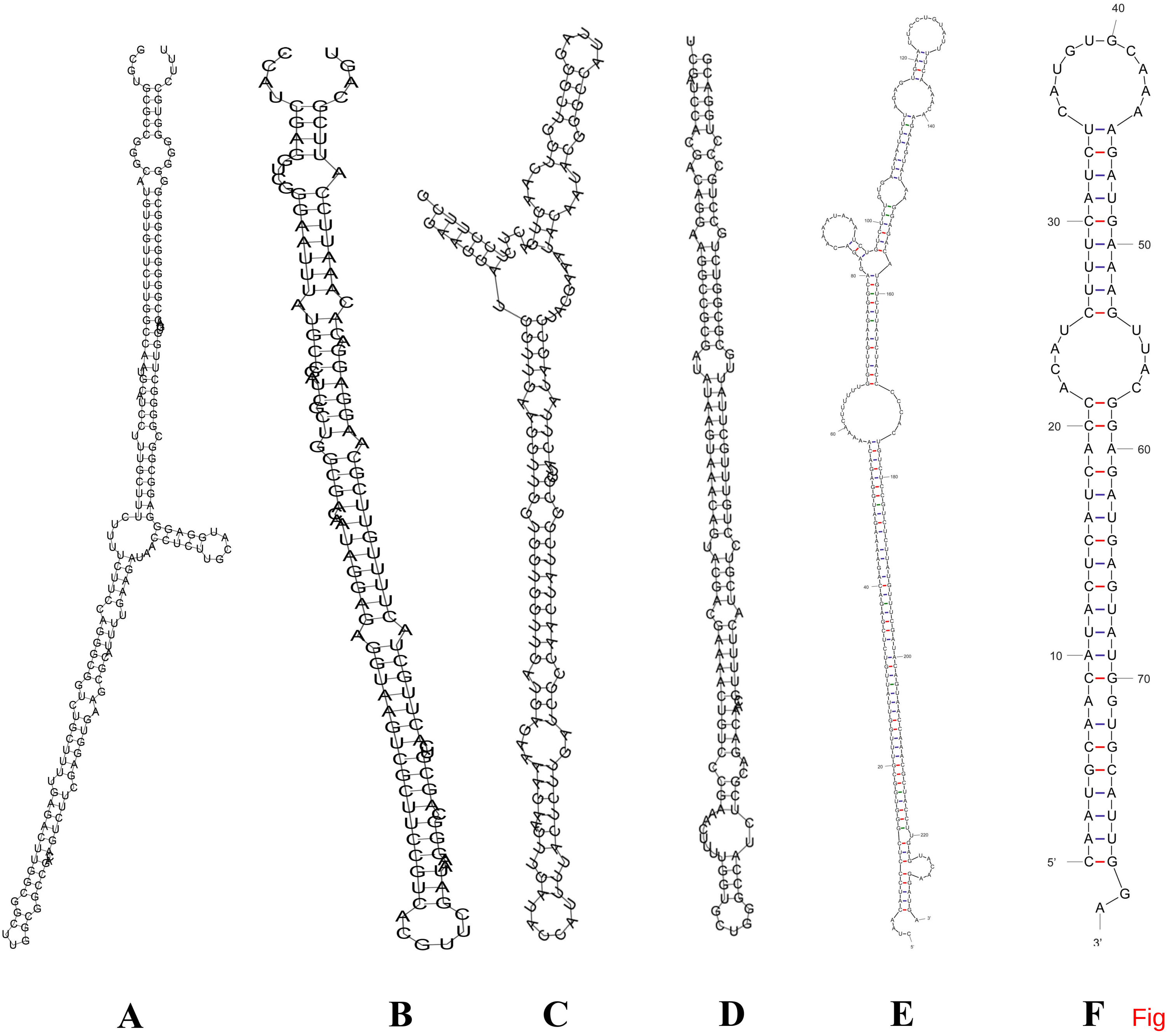

Fig. S2

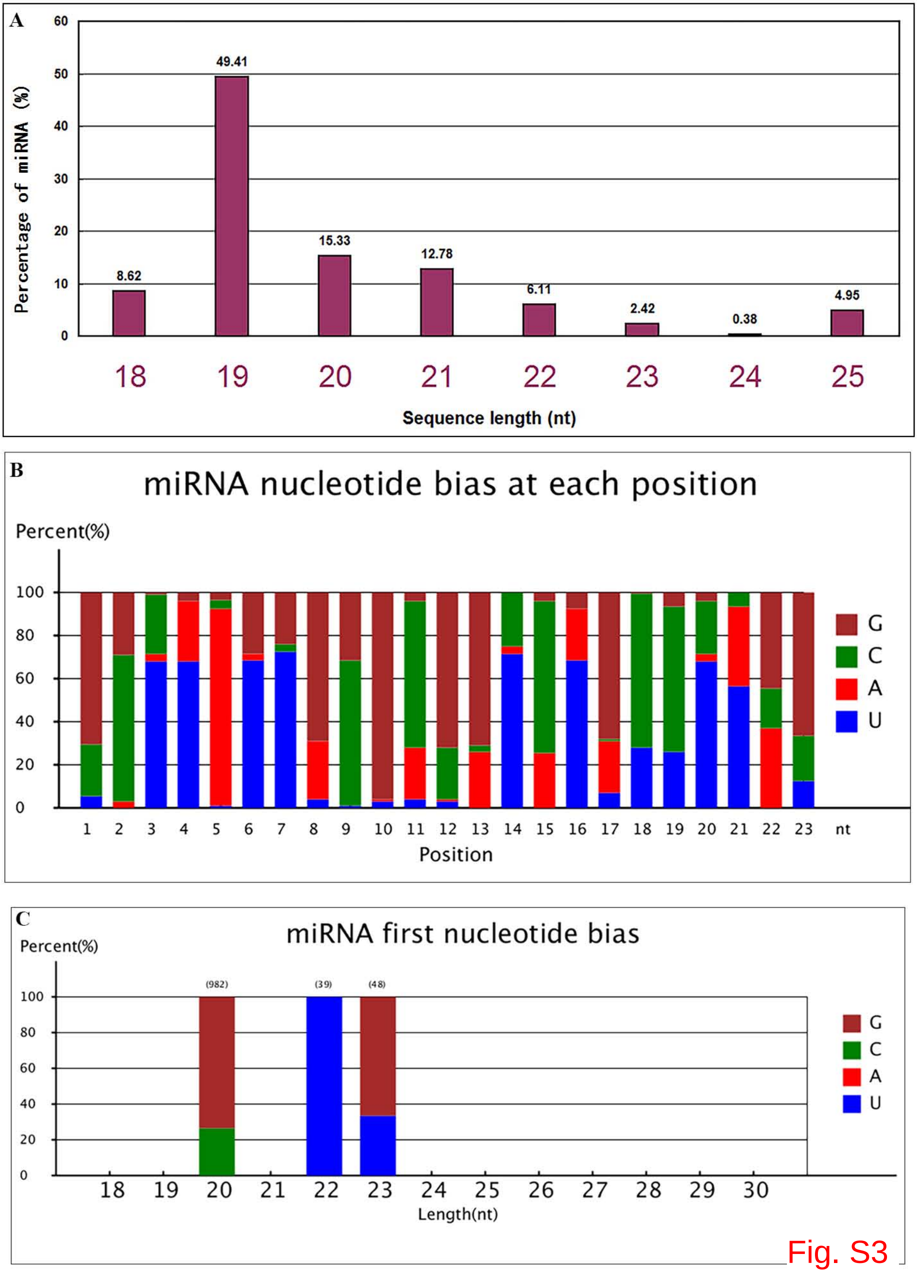
A
Percentage of miRNA (%)
60
50
40
30
20
10
0
8.62
49.41
15.33
12.78
6.11
2.42
0.38
4.95
18
19
20
21
22
23
24
25
Sequence length (nt)
B
miRNA nucleotide bias at each position
Percent(%)
100
80
60
40
20
0
1 2 3 4 5 6 7 8 9 10 11 12 13 14 15 16 17 18 19 20 21 22 23 nt
Position
G
C
A
U
C
miRNA first nucleotide bias
Percent(%)
(982)
(39)
(48)
100
80
60
40
20
0
18 19 20 21 22 23 24 25 26 27 28 29 30
Length(nt)
G
C
A
U

Fig. S3

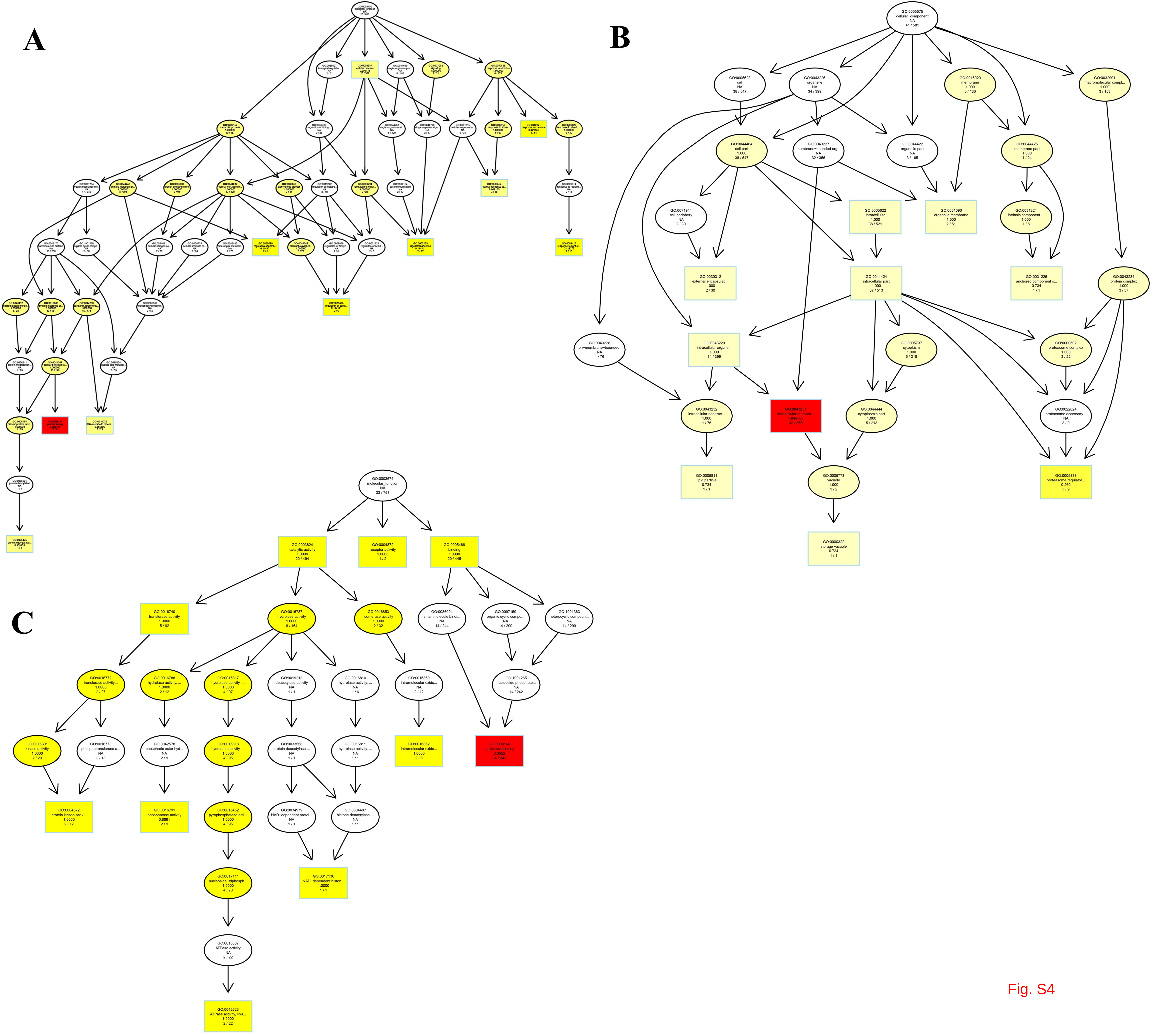
A
B
GO:0005575
cellular_component
NA
41 / 581
GO:0005623
cell
NA
38 / 547
GO:0043226
organelle
NA
34 / 399
GO:0016020
membrane
1.000
5 / 130
GO:0032991
macromolecular compl...
1.000
3 / 153
GO:0044464
cell part
1.000
38 / 547
GO:0043227
membrane−bounded org...
NA
32 / 356
GO:0044422
organelle part
NA
3 / 165
GO:0044425
membrane part
1.000
1 / 24
GO:0071944
cell periphery
NA
2 / 30
GO:0005622
intracellular
1.000
38 / 521
GO:0031090
organelle membrane
1.000
2 / 51
GO:0031224
intrinsic component ...
1.000
1 / 8
GO:0030312
external encapsulati...
1.000
2 / 30
GO:0044424
intracellular part
1.000
37 / 513
GO:0031225
anchored component o...
0.734
1 / 1
GO:0043234
protein complex
1.000
3 / 57
GO:0043228
non−membrane−bounded...
NA
1 / 76
GO:0043229
intracellular organe...
1.000
34 / 399
GO:0005737
cytoplasm
1.000
5 / 218
GO:0000502
proteasome complex
1.000
3 / 22
GO:0043232
intracellular non−me...
1.000
1 / 76
GO:0043231
intracellular membra...
1.04e−07
32 / 355
GO:0044444
cytoplasmic part
1.000
5 / 213
GO:0022624
proteasome accessory...
NA
3 / 9
GO:0005811
lipid particle
0.734
1 / 1
GO:0005773
vacuole
1.000
1 / 2
GO:0005838
proteasome regulator...
0.260
3 / 9
GO:0000322
storage vacuole
0.734
1 / 1
C
GO:0003674
molecular_function
NA
33 / 753
GO:0003824
catalytic activity
1.0000
20 / 494
GO:0004872
receptor activity
1.0000
1 / 2
GO:0005488
binding
1.0000
20 / 445
GO:0016740
transferase activity
1.0000
5 / 92
GO:0016787
hydrolase activity
1.0000
8 / 164
GO:0016853
isomerase activity
1.0000
2 / 32
GO:0036094
small molecule bindi...
NA
14 / 244
GO:0097159
organic cyclic compo...
NA
14 / 299
GO:1901363
heterocyclic compoun...
NA
14 / 299
GO:0016772
transferase activity...
1.0000
2 / 27
GO:0016788
hydrolase activity, ...
1.0000
2 / 12
GO:0016817
hydrolase activity, ...
1.0000
4 / 97
GO:0019213
deacetylase activity
NA
1 / 1
GO:0016810
hydrolase activity, ...
NA
1 / 6
GO:0016860
intramolecular oxido...
NA
2 / 12
GO:1901265
nucleoside phosphate...
NA
14 / 242
GO:0016301
kinase activity
1.0000
2 / 20
GO:0016773
phosphotransferase a...
NA
2 / 13
GO:0042578
phosphoric ester hyd...
NA
2 / 9
GO:0016818
hydrolase activity, ...
1.0000
4 / 96
GO:0033558
protein deacetylase ...
NA
1 / 1
GO:0016811
hydrolase activity, ...
NA
1 / 1
GO:0016862
intramolecular oxido...
1.0000
2 / 8
GO:0000166
nucleotide binding
0.0852
14 / 242
GO:0004672
protein kinase activ...
1.0000
2 / 12
GO:0016791
phosphatase activity
0.8861
2 / 9
GO:0016462
pyrophosphatase acti...
1.0000
4 / 95
GO:0034979
NAD−dependent protei...
NA
1 / 1
GO:0004407
histone deacetylase ...
NA
1 / 1
GO:0017111
nucleoside−triphosph...
1.0000
4 / 78
GO:0017136
NAD−dependent histon...
1.0000
1 / 1
GO:0016887
ATPase activity
NA
2 / 22
GO:0042623
ATPase activity, cou...
1.0000
2 / 22

Fig. S4

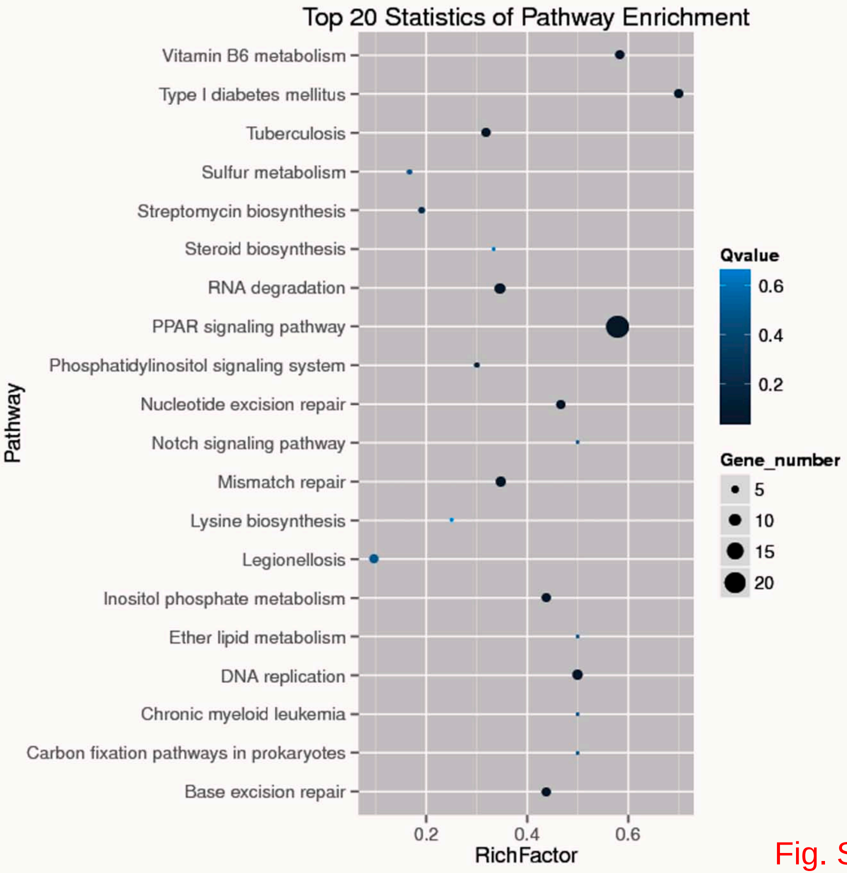
Top 20 Statistics of Pathway Enrichment
Vitamin B6 metabolism
Type I diabetes mellitus
Tuberculosis
Sulfur metabolism
Streptomycin biosynthesis
Steroid biosynthesis
RNA degradation
PPAR signaling pathway
Phosphatidylinositol signaling system
Nucleotide excision repair
Notch signaling pathway
Mismatch repair
Lysine biosynthesis
Legionellosis
Inositol phosphate metabolism
Ether lipid metabolism
DNA replication
Chronic myeloid leukemia
Carbon fixation pathways in prokaryotes
Base excision repair
Pathway
0.2
0.4
0.6
RichFactor
Qvalue
0.6
0.4
0.2
Gene_number
5
10
15
20

Fig. S5

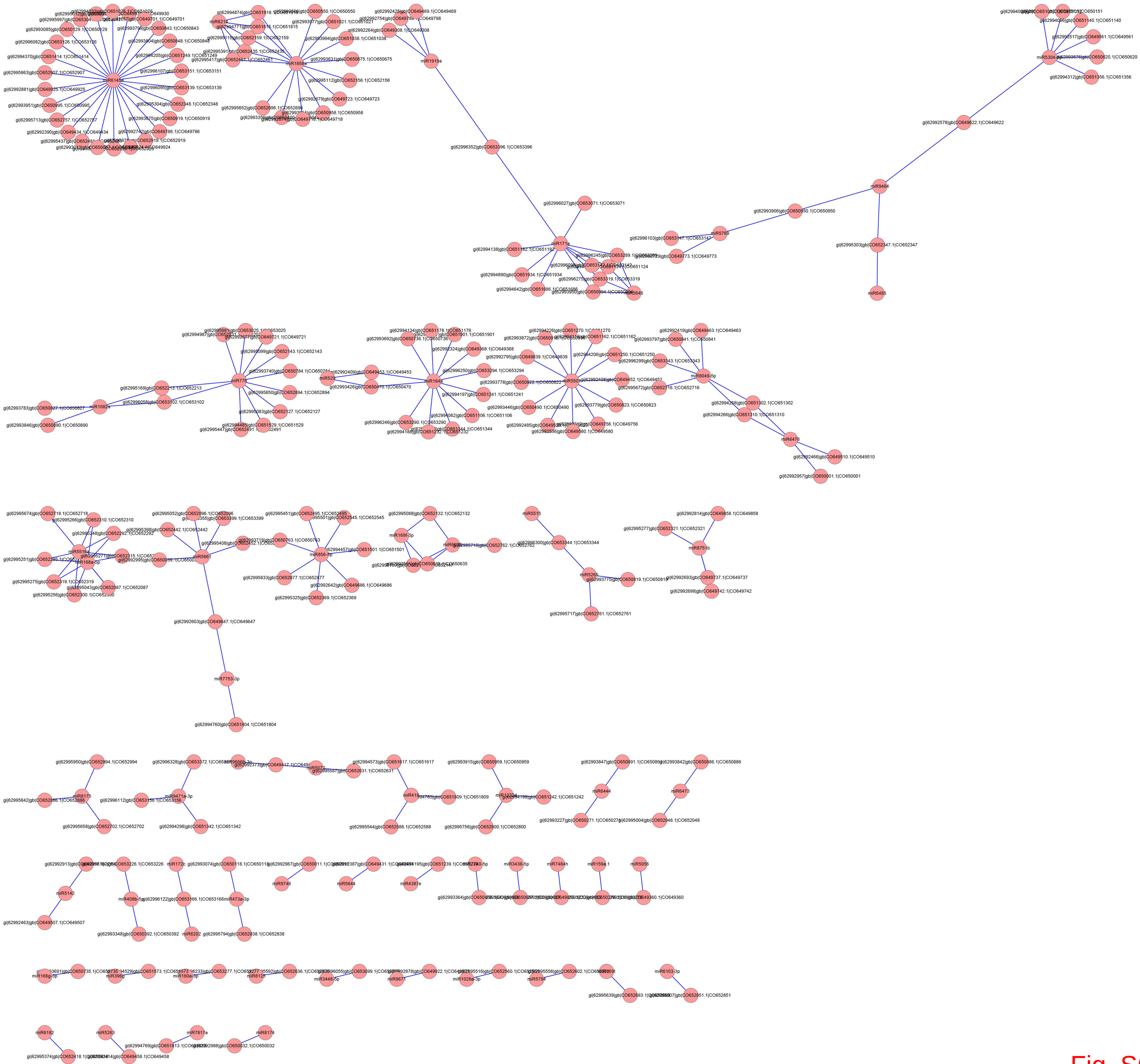

Fig. S6

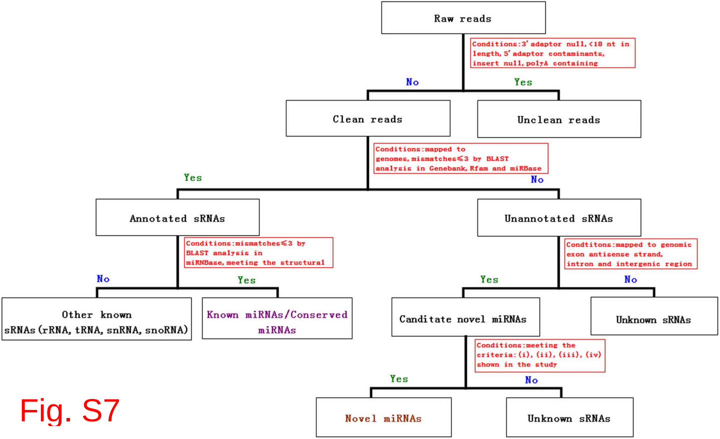

Raw reads
Conditions:3' adaptor null,<18 nt in length,5' adaptor contaminants, insert null,polyA containing
No
Yes
Clean reads
Unclean reads
Conditions:mapped to genomes,mismatches≤3 by BLAST analysis in Genebank,Rfam and miRBase
Yes
No
Annotated sRNAs
Unannotated sRNAs
Conditions:mismatches≤3 by BLAST analysis in miRNBase,meeting the structural
Condtitons:mapped to genomic exon antisense strand, intron and intergenic region
No
Yes
Yes
No
Other known sRNAs(rRNA, tRNA, snRNA, snoRNA)
Known miRNAs/Conserved miRNAs
Canditate novel miRNAs
Unknown sRNAs
Conditions:meeting the criteria:(i),(ii),(iii),(iv) shown in the study
Yes
No
Novel miRNAs
Unknown sRNAs

Fig. S7
